# Increased reproducibility in RV mass measurement at end-systole

**DOI:** 10.1186/1532-429X-17-S1-Q40

**Published:** 2015-02-03

**Authors:** Stephan P Altmayer, Laurens A Teeuwen, Robert C Gorman, Yuchi Han

**Affiliations:** 1 Cardiology, University of Pennsylvania, Philadelphia, PA, USA

## Background

Cardiovascular magnetic resonance (CMR) has been shown to be accurate and reproducible for right ventricular (RV) function evaluation. RV mass, assessed in end-diastolic (ED) phase, is one of the least reproducible CMR derived variables. The end-systolic (ES) phase could offer an alternative way to improve reproducibility, since the selection of basal slice and visualization of the usually thin RV wall are easier in this phase. Our goal was to evaluate the accuracy, reproducibility and contouring time for both ED and ES segmentation of the RV.

## Methods

Clinical CMR exams were performed on a 1.5T Siemens scanner (Avanto, Siemens Health Systems, Germany) using a steady-state free precession (SSFP) sequence (flip angle = 55-75°, TR = 25-35 ms, TE = 1.0-1.5 ms, 30 cardiac phases, slice thickness of 8 mm). Animal CMR were performed on a 3T Siemens scanner (Trio, Siemens Health Systems, Germany) using a SSFP sequence (flip angle = 45°, TR = 25-30 ms, TE = 1.5 ms, 60 images per cardiac cycle, slice thickness of 4 mm). Two observers - Observer 1 (Ob1), 6-months experienced, and Observer 2 (Ob2) with no previous experience - received practical training before the study began. RV mass and volumes were obtained in ES and ED phases using QMASS (Medis, Leiden, The Netherlands). To evaluate accuracy, 7 sheep hearts were imaged in vivo and then their RV free wall were weighted after removing epicardial fat. To assess reproducibility in clinical scans, Ob1 processed ventricular function of a 30 normal subjects. After at least 3 weeks, Ob1 and Ob2 reprocessed 15 randomly selected subjects. Absolute variability, Coefficient of variation (CoV) and intraclass correlation coefficient (ICC) were used to evaluate intra and inter-observer variability. Segmentation time was recorded after the visual selection of ES and ED phases.

## Results

Both phases were accurate to predict the weighted RV mass in animals (Table 1). ES presented less absolute variability (5.6% ±3.6) than ED (8.7% ±3.9), despite not statistically significant (p = 0.14). The intra- and inter-observer reproducibility was higher in the ES phase for normal subjects. RV mass measured in the ED phase always yielded higher absolute variability and CoV than the ES phase (Table 2). Mean segmentation time (minutes ±SD) was significantly lower (p<0.001) in the ES than ED (4.9 ±1.1 vs. 6.6 ±1.2).

**Table 1:** Weighted and CMR-derived RV mass (grams) in animal studies

	Weighted mass	End-diastolic phase		End-systolic phase	
		
		Observer 1	Observer 2	Observer 1	Observer 2
Sheep 1	34.4	30.75	28.7	32.45	30.3

Sheep 2	49.1	46.65	59.2	50.2	54.55

Sheep 3	38.85	35.55	39.65	40.5	36.95

Sheep 4	26.2	26.05	22.85	25.9	23.95

Sheep 5	57.6	50.6	49.1	52.75	50.45

Sheep 6	32.75	32.2	37.35	32.6	31.65

Sheep 7	32.4	28.85	29.2	30.45	30.25

Mean ±SD (p-value)	38.7 ±10.9	37.35 ±10.8 (0.26)		36.9 ±10.8 (0.24)	

Absolute Variability		5.6% ±3.5		8.7% ±3.9	

**Table 2:** Intra- and inter-observer variability of CMR-derived RV mass in normal subjects

	Intra-observer			Inter-observer		
	
	Absolute Variability	CoV	ICC	Absolute Variability	CoV	ICC
Diastole	12.6% ±4.1	9.1%	0.88	18.6% ±9.7	14.9%	0.66

Systole	6.6% ±5.1	5%	0.93	12.2% ±7.8	7.9%	0.84

CoV, Coefficient of variation; ICC, Intraclass correlation coefficient.

## Conclusions

The assessment of RV mass is accurate, more reproducible and faster in ES phase.

## Funding

Cardiovascular Medical Research and Education Fund.

